# Oculoleptomeningeal Amyloidosis associated with transthyretin Leu12Pro in an African patient

**DOI:** 10.1007/s00415-014-7594-2

**Published:** 2014-12-09

**Authors:** P. McColgan, S. Viegas, S. Gandhi, K. Bull, R. Tudor, F. Sheikh, J. Pinney, M. Fontana, D. Rowczenio, J. D. Gillmore, J. A. Gilbertson, C. J. Whelan, S. Shah, Z. Jaunmuktane, J. L. Holton, J. M. Schott, D. J. Werring, P. N. Hawkins, M. M. Reilly

**Affiliations:** 1 MRC Centre for Neuromuscular Diseases, UCL Institute of Neurology, Queen Square, London, WC1N 3BG UK; 2 Cavan General Hospital, Lisdarn, Cavan, Republic of Ireland; 3 National Amyloidosis Centre, Royal Free Hospital, Rowland Hill Street, London, NW3 2PF UK; 4 Department of Neuroradiology, National Hospital of Neurology and Neurosurgery, University College London Hospitals, Queen Square, London, WC1N 3BG UK

**Keywords:** Genetics, Amyloid, Transthyretin, Leu12Pro

## Abstract

Oculoleptomeningeal amyloidosis is a rare manifestation of hereditary transthyretin (TTR) amyloidosis. Here, we present the first case of leptomeningeal amyloidosis associated with the TTR variant Leu12Pro mutation in an African patient. A 43-year-old right-handed Nigerian man was referred to our centre with rapidly progressive neurological decline. He presented initially with weight loss, confusion, fatigue, and urinary and erectile dysfunction. He then suffered recurrent episodes of slurred speech with right-sided weakness. He went on to develop hearing difficulties and painless paraesthesia. Neurological examination revealed horizontal gaze-evoked nystagmus, brisk jaw jerk, increased tone, brisk reflexes throughout and bilateral heel-shin ataxia. Magnetic resonance imaging showed extensive leptomeningeal enhancement. Cerebrospinal fluid analysis showed a raised protein of 6.4 g/dl. Nerve conduction studies showed an axonal neuropathy. Echocardiography was characteristic of cardiac amyloid. TTR gene sequencing showed that he was heterozygous for the leucine 12 proline mutation. Meningeal and brain biopsy confirmed widespread amyloid angiopathy. TTR amyloidosis is a rare cause of leptomeningeal enhancement, but should be considered if there is evidence of peripheral or autonomic neuropathy with cardiac or ocular involvement. The relationship between different TTR mutations and clinical phenotype, disease course, and response to treatment remains unclear.

## Introduction

Transthyretin (TTR), a serum precursor protein of amyloid, [1] is predominantly produced in the liver. However, some TTR is also produced in the central nervous system (CNS) within the choroid plexus and the pigmented epithelium of the retina [2]. Many mutations in the transthyretin gene are known to cause familial amyloid polyneuropathy (FAP), an autosomal dominant multisystem disease [3].

OLMA is a rare manifestation of TTR amyloidosis, characterised by amyloid deposition in the meninges of the brain and spinal cord, often with ocular involvement. Patients present with a wide variety of neurological problems including epilepsy [4], subarachnoid haemorrhage [5], hearing or visual loss [6, 7] and headache [8]. While many TTR mutations can cause both FAP and OLMA [6, 9, 10], the extremely rare Leu12Pro mutation has only been associated with OLMA to date.

We described the first case of the TTR Leu12Pro mutation in 1999 [8]. To date, the only cases described have been in patients of European ancestry. Here, we present the first case of the TTR Leu12Pro variant in a patient of African origin.

## Case report

A 43-year-old right-handed Nigerian man was referred to our centre with rapidly progressive neurological decline over a 19-month period. He presented initially in March 2011 with weight loss of 20 kg over 3 months. Two months later, he developed progressive confusion, fatigue, erectile difficulties, urinary urgency and incontinence. In March 2012, he suffered from recurrent episodes of slurred speech and right-sided weakness, lasting 30 min. At his local hospital, a non-contrast MRI head was normal, but an echocardiogram identified a restrictive cardiomyopathy and he was started on aspirin. The episodes were initially every 2 weeks but increased in frequency over time. In June 2012, he developed new left-sided hearing difficulties. By October 2012, he had developed painless paraesthesia in both feet.

There was no relevant past medical history. His father had suffered from weight loss, erectile dysfunction and renal problems, and died of a myocardial infarction in his 60s. His mother died in her 50s of unknown cause. Of his four siblings, the oldest brother died at age 14 but no clear cause was established. His sister had developed deafness in her 40s.

Cardiovascular, respiratory and abdominal examination was unremarkable. Screening cognitive testing demonstrated anterograde memory difficulties, and impairment of attention and concentration; the MMSE score was 21/30. He had horizontal gaze-evoked nystagmus and a brisk jaw jerk, but cranial nerve examination was otherwise normal. Neuro-ophthalmological assessment was normal. Examination of the limbs revealed increased tone and brisk reflexes in the upper and lower limbs and mild bilateral heel-shin ataxia.

## Investigations

The following blood tests were normal or negative: Full blood count, urea and electrolytes, liver function tests, thyroid function, cardiac enzymes, B12, folate, serum ACE, HIV, HTLV-1, and treponemal serology, ANA, ANCA, anti-neuronal antibodies, serum-free light chain assay, electrophoresis and immunofixation. Urine testing confirmed a normal creatinine clearance and no proteinuria. On the basis of the autonomic symptoms and restrictive cardiomyopathy, sequencing of the TTR gene was performed, revealing that he was heterozygous for the mutation leucine 12 proline.

## Neurological investigations

**Fig. 1:**
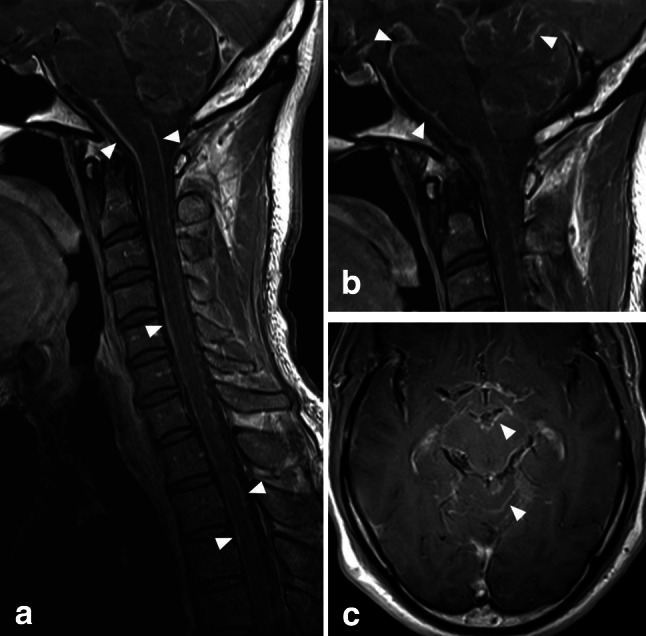
Post-gadolinium T1-weighted MR images of the cervical spine (**a**, **b**) and brain (**c**), demonstrating diffuse, mildly nodular leptomeningeal enhancement over the surface of the spinal cord, brain stem and cerebellum (*arrowheads*)

**Fig. 2:**
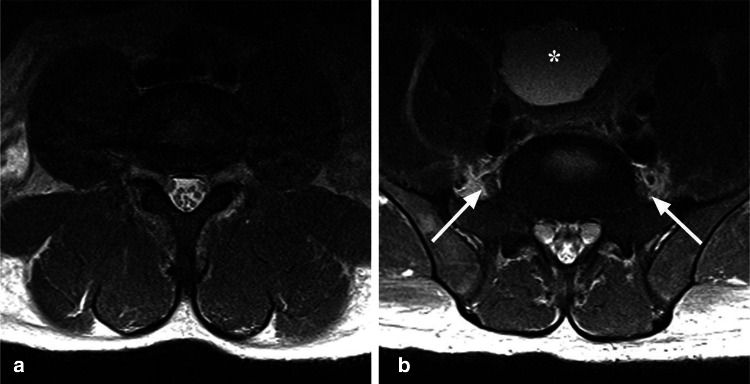
Axial T2-weighted MR images of the lumbosacral spine (**a**, **b**), demonstrating mild hypertrophy of the spinal nerve roots. Mild prominence of the lumbosacral plexus bilaterally is also shown (*arrows*). Note the distended urinary bladder with a trabeculated wall (*asterisk*)

Magnetic resonance imaging (MRI) of the brain and spine showed extensive leptomeningeal enhancement over the surface of the brain and spinal cord (Fig. 1). No signal change was demonstrated within the underlying parenchyma. Susceptibility-weighted sequences revealed no parenchymal microhaemorrhages, or evidence of subarachnoid blood products. Magnetic resonance angiography of the intra- and extra-cranial arteries was normal. There was also evidence of mild, diffuse thickening of the intrathecal nerve roots, and nerves of the brachial and lumbosacral plexus (Fig. 2).

Cerebrospinal fluid (CSF) analysis showed seven white cells (four lymphocytes and three polymorphs), CSF protein was elevated at 6.4 g/dl, with a normal glucose ratio (3.6 mmol/l CSF and 5.9 mmol/l serum), oligoclonal bands were not detected. Whilst the CSF Ab1-42 was depressed (371 pg/ml, reference range 627–1,322) possibly reflecting sequestration within CNS amyloid plaques as is seen in Alzheimer’s disease (AD), unlike in AD the CSF tau level was also low (107 pg/ml, reference range 146–595) mitigating against an ongoing neurodegenerative process. Two further CSF examinations showed a mild pleocytosis and persistently raised CSF protein. No organisms were cultured (including mycobacterium tuberculosis) and no cellular atypia was seen.

Nerve conduction studies demonstrated a mild large fibre peripheral neuropathy in the lower limbs and a severe small fibre neuropathy affecting both the upper and lower limbs. Autonomic function testing showed a low resting supine blood pressure (BP) of 101/65 mmHg and orthostatic hypotension on standing at 3 min with BP dropping to 77/50 mmHg. A 48-h period of inpatient video telemetry showed mild global cortical dysfunction but no focal features or epileptiform abnormalities.

Neuro-otology investigations confirmed a mixed left-sided peripheral and central vestibular disorder with sparing of the auditory pathway. Neuropsychometry testing showed profoundly impaired processing speed with widespread cognitive impairment reflecting severe subcortical dysfunction.

**Fig. 3:**
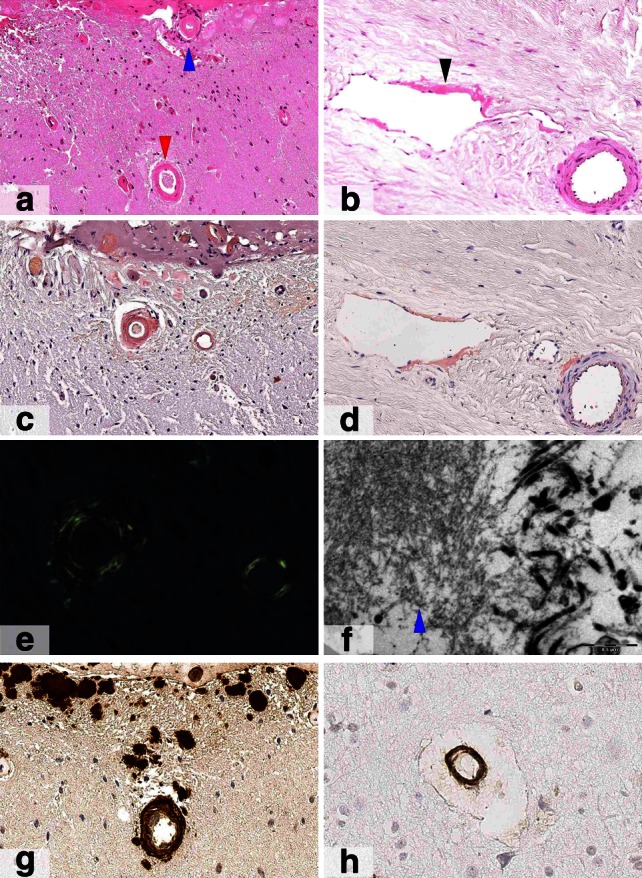
Brain biopsy of meningeal and cerebral amyloid angiopathy Haematoxylin-Eosin stained sections (**a** and **b**) show thickened brightly eosinophilic walls of small vessels in the leptomeninges (*blue arrowhead* in **a**), neocortex (*red arrowhead* in **a**) and pachymeninges (*black arrowhead* in **b**). Affected vessels are positively stained with Congo red (**c** and **d**) which shows apple green birefringence under polarised light (**e**). Ultrastructural assessment (**f**) confirms the presence of haphazardly arranged amyloid fibrils in the dura mater (*blue arrowhead*). TTR protein (G) is widely deposited in the walls of the blood vessels and in addition shows patchy parenchymal deposits with a subpial distribution. A small proportion of the vessels in brain parenchyma also contain amyloid β deposits (H) likely to represent co-localization of entrapped amyloid β protein. *Scale bar* 100 µm (**a**–**d**); 30 µm (**e**); 0.5 µm (**f**); 50 µm (**g**–**h**)

A brain biopsy was performed to confirm amyloid deposition and exclude any other pathology. The histology confirmed widespread amyloid angiopathy involving dura mater, leptomeninges and neocortical blood vessels as well as occasional vessels in the white matter. There was no evidence of underlying malignancy or vasculitis. (see Fig. 3).

## Other investigations

Serum amyloid P component scintigraphy demonstrated the modest amyloid deposits in the spleen and kidneys.

ECG revealed sinus rhythm with small complexes in the inferior leads. Echocardiogram demonstrated an ejection fraction of 51 %, mild to moderate diastolic dysfunction with elevated filling pressures. Cardiac MRI showed moderate left ventricular (LV) wall thickening and moderate LV systolic dysfunction, along with diffuse biventricular late gadolinium enhancement typical of cardiac amyloidosis. TC-labelled DPD scintigraphy showed grade III localization in the heart, characteristic of cardiac TTR amyloidosis.

Arterial blood gas sampling demonstrated central hypoventilation causing respiratory failure.

## Disease course

During his inpatient stay he continued to deteriorate, remaining very drowsy with fluctuating agitation and confusion. Liver transplantation was considered but excluded due to the lack of evidence for benefiting TTR amyloidosis patients with CNS disease, along with the presence of cardiac amyloidosis and the rapid deterioration of the patient’s cognitive function. He was subsequently repatriated to his local hospital. At the time of writing, he has spent 330 days in hospital. There has been a gradual decline in his overall condition. He has developed an atonic bladder, with recurrent urosepsis and now has an indwelling catheter.

## Discussion

OLMA was first described by Goren in 1980 [11], since then 14 TTR mutations causing leptomeningeal amyloidosis have been identified [5, 7, 9–16]. Until now, these have been found exclusively in European, American and Japanese patients. The syndrome remains extremely rare, with only 74 patients reported with OLMA in the literature [4–30]. Age of onset ranges from 28 to 69 years, with a large variation in time from presentation to time of death, ranging from 6 months to 26 years (Table 1). The reported cases of Leu12Pro have presented with a spectrum of cognitive difficulties, ataxia, epilepsy, headache, peripheral neuropathy, autonomic dysfunction, visual and hearing loss. We report here the first case of leptomeningeal amyloidosis in an African patient with the Leu12Pro mutation. This patient presented with episodic symptoms of dysarthria and right-sided weakness, on a background of an autonomic and peripheral neuropathy, and cognitive decline. In addition, he also demonstrated pyramidal and cerebellar signs on examination.

**Table 1:** TTR mutations causing leptomeningeal amyloidosis reported in the literature

Author	Garzuly	Jin	Salvi	Shimizu	Hagiwara	Llul	Douglass	Liepnieks	Ellie	Barreiros^a^	Urban^a^	Brett	Current	Uemichi	Klein
Mutation	A18G	A18G	A36P	A25T	A25T	A25T	G53A	G53A	G53G	L12P	L12P	L12P	L12P	P64S	Ser44
Cases	4	2	1	1	1	1	1	2	3	2	1	1	1	3	1
Country	Hungary	Japanese	Italy	Japanese	Japanese	Spanish	British	American	France	German	German	British	Nigerian	Canada	Irish
Age at presentation (years)	36–53	40–42	69	41	52	53	44	48–56	42–46	28–37	31	38	42	28–31	32
Cognitive	✔	✖	✔	✖	✖	✖	✔	✔	✔	✔	✔	✔	✔	✔	✖
Ataxia	✔	✔	✔	✔	✔	✔	✖	✖	✔	✖	✖	✔	✔	✔	✖
Epilepsy	✖	✖	✖	✖	✔	✖	✖	✖	✖	✔	✔	✔	✖	✔	✖
Headache	✔	✔	✖	✖	✖	✖	✔	✖	✔	✖	✖	✔	✔	✔	✔
Autonomic	✔	✔	✖	✖	✖	✖	✔	✖	✔	✖	✖	✔	✔	✔	✖
Peripheral Neuropathy	✖	✖	✖	✔	✔	✖	✔	✖	✖	✖	✖	✔	✔	✔	✔
Hearing loss	✔	✔	✔	✔	✔	✔	✖	✖	✖	✖	✖	✔	✔	✔	✖
Visual loss	✔	✔	✔	✔	✖	✖	✖	✖	✖	✔	✔	✖	✖	✔	✔
Vascular	✖	SAH	✖	✖	IC	✖	✖	TIA	SAH	✖	✖	SAH	✖	ICH	✖
GI	✔	✖	✖	✖	✖	✖	✖	✖	✖	✖	✖	✖	✖	✖	✔
Pyramidal Signs	✔	✔	✔	✖	✖	✔	✔	✖	✔	✖	✖	✖	✔	✔	✖
Imaging	LME	LME/SS	SS	LME	LME	SS	LME	LME/HC	LME	–	LME	LME/HC	LME	–	–
CSF Protein	High/XC	High	–	High	High/XC	High/XC	–	N/A	High	–	High/XC	high	High/XC	High	–
CNS Amyloid	✔	✔	✔	✔	✔	✔	–	✔	✔	–	–	✔	✔	✔	–
Transplant	✖	✖	✖	✖	✖	✖	✖	✖	✖	✔ (2)	✔ (1)	✖	–	✖	–
Disease duration	7–22 years	–	–	–	–	–	–	17 years	1–2 years	6 months–3 years	–	15 years	–	1–12 years	–

Author	Hirai	Nakagawa	Suhr	Schweitzer	Blevins	Roe	Dowell	Petersen	Herrick	Furuya	Nakamaura	Horowitz	Goren	Taratuto
Mutation	T114C	T49P	T69H	T69H	T69H	V30G	V30G	V30G	V30M	V30M	Y114C	–	–	–
Cases	3	1	1	3	7	1	1	6	1	10	8	1	7	1
Country	Japanese	Ireland	Sweden	Canada	Sweden	American	German	German	Mexican	Japanese	Japanese	American	American	Basque
Age at presentation (years)	40–52	53	58	51	37–68	44	45	46–56	69	36–58	30–53	35	35–57	52
Cognitive	✔	✔	✔	✔	✔	✖	✖	✔	✔	✖	✔	✖	✔	✖
Ataxia	✖	✖	✖	✖	✔	✖	✖	✔	✖	✔	✔	✖	✔	✖
Epilepsy	✖	✖	✔	✔	✔	✔	✔	✔	✖	✖	✖	✖	✔	?
Headache	✖	✔	✖	✔	✖	✖	✔	✖	✔	✖	✖	✖	✔	✖
Autonomic	✔	✖	✖	✖	✖	✖	✖	✖	✔	✔	✔	✔	✔	✖
Peripheral neuropathy	✔	✔	✔	✖	✔	✖	✖	✔	✔	✔	✔	✔	✔	✖
Hearing loss	✖	✖	✔	✖	✖	✖	✖	✖	✖	✖	✖	✖	✔	✔
Visual loss	✖	✖	✖	✖	✔	✔	✖	✔	✖	✔	✔	✖	✔	✖
Vascular	SAH	✖	✖	✖	SLE	✖	✖	✖	✖	✖	SLE	✖	✖	?
GI	✖	✖	✖	✖	✖	✖	✔	✖	✖	✖	✖	✖	✔	✖
Pyramidal Signs	✔	✖	✖	✔	✔	✖	✖	✖	✔	✔	✔	✖	✔	?
Imaging	LME	LME	LME	LME	LME	LME	LME	LME	LME	–	LME	LME	–	LME
CSF Protein	N/A	High	N/A	High	High	High	High	N/A	High	–	High	–	High	High
CNS amyloid	–	✔	–	–	✔	✔	✔	✔	✔	✔	✔	✔	✔	✔
Transplant	✔ (2)	✖	✖	✔ (1)	✔ (1)	✖	✖	✖	✖	✖	✖	✖	✖	✖
Disease duration (year)	3–5	–	–	6	1–8	–	3	3–26	–	–	–	–	2–9	–

*LME* leptomeningeal enhancement, *SS* superficial siderosis, *HC* hydrocephalus, *ICH* intracerebral haemorrhage, *XC* xanthochromia, *SAH* subarachnoid haemorrhage, *SLE* stroke like episodes, *TIA* transient ischaemic attack

^a^Same patient included in two papers

Leptomeningeal amyloid was most likely responsible for the transient focal neurological episodes. Convexity SAH and siderosis with TTR mutations has been described [5], and that although we did not find SAH or siderosis, leakage of blood products from amyloid laden fragile leptomeningeal small vessels may have been the underlying substrate for the attacks. Such transient attacks have been described frequently in sporadic cerebral amyloid angiopathy [31].

The contribution of TTR produced in various anatomical sites to the disease-causing CNS amyloid deposits in OLMA remains unclear, i.e. TTR produced locally within the brain versus liver-derived TTR from the systemic circulation. Whilst de novo oculoleptomeningeal amyloid deposition apparently may occur after liver transplantation has been performed in patients with TTR mutations to remove the source of variant TTR in the blood [32], many studies have also confirmed that wild-type TTR can continue to accumulate after liver transplant on the existing template of amyloid associated with variant TTR. A study of a patient who received a domino liver transplant using an explanted FAP Val30Met patient’s liver has confirmed that mutant TTR in the plasma does enter the brain [33]. Whilst liver transplantation has been performed in patients with OLMA associated with various TTR mutations, published outcome has varied and its role remains unclear. Survival for patients with TTR Leu12Pro has ranged from 6 months to 3 years [17], while survival at 5 and 6 years was reported with T114C [21] and T69H [23] patients, respectively. Yamashita and colleagues also corroborated that patients with T114C mutation may have a better prognosis following liver transplantation. In a study of 8 patients with the T114C mutation, they found that those receiving liver transplant had reduced mortality and morbidity related to dementia and subarachnoid haemorrhage [34].

The selectivity of Leu12Pro mutations to the central nervous system is postulated to be due to regional differences in cell proteostasis. While unstable mutants, such as Leu12Pro, are subject to degradation by the endoplasmic reticulum in the liver, mutant TTR produced by the choroid plexus binds to thyroxine (T4) causing stabilisation, thereby preventing endoplasmic reticulum degradation. Once the mutant TTR is released in the CSF T4 dissociates causing destabilisation and the formation of TTR aggregates [35].

Although OLMA is a rare condition, it is a devastating and life-threatening disease. Its precise pathogenesis and the mechanism of disease progression, which are remarkably rapid in our case, remain unclear as does the role of liver transplantation.
